# Exploring synergistic interactions and catalysts in complex interventions: longitudinal, mixed methods case studies of an optimised multi-level suicide prevention intervention in four european countries (Ospi-Europe)

**DOI:** 10.1186/s12889-016-2942-z

**Published:** 2016-03-15

**Authors:** Fiona M. Harris, Margaret Maxwell, Rory O’Connor, James C. Coyne, Ella Arensman, Claire Coffey, Nicole Koburger, Ricardo Gusmão, Susana Costa, András Székely, Zoltan Cserhati, David McDaid, Chantal van Audenhove, Ulrich Hegerl

**Affiliations:** Nursing, Midwifery & Allied Health Professions Research Unit, University of Stirling, Unit 13, University of Stirling Innovation Park, Stirling, FK9 4NF UK; Institute of Health & Well-being, University of Glasgow, Glasgow, UK; Faculty of Medical Sciences, University of Groningen, Groningen, The Netherlands; National Suicide Research Foundation, University College Cork, Cork, Ireland; Department of Psychiatry and Psychotherapy, University of Leipzig, Leipzig, Germany; CEDOC, Faculdade e Ciências Médicas, Universidade Nova de Lisboa, Lisbon, Portugal; Institute of Behavioural Sciences, Semmelweis University Budapest, Budapest, Hungary; Personal Social Services Research Unit, London School of Economics and Political Science, London, UK; LUCAS, KU Leuven, Leuven, Belgium

**Keywords:** Complex interventions, Longitudinal study, Process evaluation, Suicide prevention, Synergistic interactions, Programme as catalyst

## Abstract

**Background:**

The Medical Research Council (MRC) Framework for complex interventions highlights the need to explore interactions between components of complex interventions, but this has not yet been fully explored within complex, non-pharmacological interventions. This paper draws on the process evaluation data of a suicide prevention programme implemented in four European countries to illustrate the synergistic interactions between intervention levels in a complex programme, and to present our method for exploring these.

**Methods:**

A realist evaluation approach informed the process evaluation, which drew on mixed methods, longitudinal case studies. Data collection consisted of 47 semi-structured interviews, 12 focus groups, one workshop, fieldnoted observations of six programme meetings and 20 questionnaires (delivered at six month intervals to each of the four intervention sites). Analysis drew on the framework approach, facilitated by the use of QSR NVivo (v10). Our qualitative approach to exploring synergistic interactions (QuaSIC) also developed a matrix of hypothesised synergies that were explored within one workshop and two waves of data collection.

**Results:**

All four implementation countries provided examples of synergistic interactions that added value beyond the sum of individual intervention levels or components in isolation. For instance, the launch ceremony of the public health campaign (a level 3 intervention) in Ireland had an impact on the community-based professional training, increasing uptake and visibility of training for journalists in particular. In turn, this led to increased media reporting of OSPI activities (monitored as part of the public health campaign) and also led to wider dissemination of editorial guidelines for responsible reporting of suicidal acts. Analysis of the total process evaluation dataset also revealed the new phenomenon of the OSPI programme acting as a catalyst for externally generated (and funded) activity that shared the goals of suicide prevention.

**Conclusions:**

The QuaSIC approach enabled us to develop and refine our definition of synergistic interactions and add the innovative concept of catalytic effects. This represents a novel approach to the evaluation of complex interventions. By exploring synergies and catalytic interactions related to a complex intervention or programme, we reveal the added value to planned activities and how they might be maximised.

## Background

Unlike pharmacological interventions that often involve testing a single drug against controls, a complex intervention is so-called because in order to deliver it, it may require a number of elements to be put in place in order to facilitate intervention delivery [1]. Four years ago, authors of the revised Medical Research Council (MRC) Framework identified the need to understand how components or levels of a complex intervention interact [2–4], yet this remains relatively unexplored. This paper therefore explores interactions between components within a complex intervention as well as the synergistic effects that arise from these interactions. These issues are explored with respect to a multi-level suicide prevention intervention in four European countries (OSPI-Europe). Because many suicide prevention strategies are complex and multi-faceted, it is difficult to identify definitively which components might be most effective and there is little evidence of what works [5, 6] to prevent suicidal behaviour. A greater understanding of any synergistic interactions or synergies between components of multi-level interventions could inform strategies to achieve a greater reduction in deaths by suicide. More generally, gaining an understanding of how planned intervention activities might act as catalysts to generate related, external actions that share the intervention goals may allow researchers to plan ways to maximise the potential to accrue added value to planned activities.

A key aspect of complex interventions is the fact that, as complex systems, they are more than the sum of their parts [7, 8] and thus borrowing from Oakley [8] we define synergistic effects as occurring *‘where the combined effect of two (or more) intervention components is greater than the sum of the two parts provided in isolation’.* Synergistic effects between drug treatments are well researched. An example of this is provided by a systematic review of combined drug treatment for Bell’s Palsy [9], which found that administering both corticosteroids and anti-viral agents together was more effective than the sum of the effect sizes of treatment with a single drug. However, the same cannot be said for non-pharmacological, complex interventions.

Identifying synergistic effects becomes more complex when interventions aim to affect changes in human behaviour at population rather than individual level. We can distinguish between a ‘simple’ complex intervention (such as individual psychotherapy) and a community-based intervention where the multiple, often simultaneously implemented, levels of intervention might better be described as a complex ‘programme’. In the latter, the context for intervention plays a crucial role in either facilitating or reducing opportunities for synergies between different levels of intervention. Furthermore, it is important to explore how different levels of an intervention or indeed the programme as a whole may act as a catalyst that generates further interventions external to planned programme activities yet sharing common goals and contributing to positive outcomes.

OSPI-Europe has five levels of interventions targeting suicide prevention. These include training for primary care (level one) and community-based (level three) professionals; a public health campaign (level two); support for patients and families (level four) and reducing access to lethal means (level five). Level One primary care training primarily focuses on training general practitioners (GPs), while the community-based professionals who received suicide awareness and prevention training included social workers, teachers, the clergy and members of emergency services such as the police, ambulance and fire fighters. The public health campaign involved public information events; the distribution of leaflets and posters aimed at raising awareness of mental health issues; and public information broadcasts via radio and cinema. Support for patients and families included support for self help groups and signposting sources of help for those at risk. Finally, level five activity was mostly restricted to identifying suicide hot spots as the four research teams did not have the power or budget to enact some of the structural changes necessary to reduce access to means of suicide. A further discussion of the OSPI-Europe approach and its’ early implementation is provided elsewhere [10, 11]. Because of the importance of context to identifying synergies between intervention levels, we explored this within a process evaluation conducted in all four study countries: Germany, Hungary, Ireland and Portugal.

The objectives of this paper therefore are to draw on process evaluation data in order to illustrate the synergistic and catalytic interactions in a complex intervention and to identify some of the added value of achieving an impact that is greater than the sum of its’ component parts.

## Methods

Each of the four research teams gained ethical review and approval from the relevant bodies in each country: Ethics Commission of the Medical Faculty, University of Leipzig, Germany (refs. 248-2007 and 140-2009-06072009); Semmelweis University Regional and Institutional Committee of Science and Research Ethics, Hungary (ref. TUKEB 149/2009), Ethics Research Committee of the Mid-West Regional Hospital, Limerick City and County, Ireland (no reference number, letter of approval dated 25/06/2009) and Clinical Research Ethics Committee, Merlin Park University Hospital, Galway City and County, Ireland (ref. C.A. 271); and the Ethical Committee of the Faculty of Medical Sciences, New University of Lisbon, Portugal (ref. CE/DP/7-2009).

The process evaluation was informed by a realist evaluation approach [12], which applied an analysis of contexts, mechanisms and outcomes to the OSPI interventions in the four implementation countries. Evidence of synergies between intervention components were identified within this analysis. The realist evaluation approach of identifying context, mechanisms and outcomes informed our development of a method to explore synergistic effects, which we call the QuaSIC approach: the qualitative approach to exploring synergistic interactions and catalyst within a complex intervention.

The process evaluation followed a longitudinal, mixed method case study design [13, 14]. Four waves of qualitative and quantitative data were collected at six monthly intervals (January 2010 – December 2011). This included semi-structured interviews (*n* = 47) and focus groups (*n* = 12) with local mental health stakeholders closely involved with implementation activities, fieldnotes recorded at six intervention team meetings, and five waves of questionnaires. The interviews and focus groups were conducted with professionals who had some ‘stake’ in suicide prevention, including health professionals (GPs, mental health nurses, psychologists, psychiatrists), community-based professionals (e.g. members of the police, social and community workers), mental health charities and mental health advocates. The questionnaires were designed to track progress with implementation (e.g in terms of content and intensity) in each of the four countries and were completed by one researcher at each of the four intervention sites. Interviews and focus groups were recorded, transcribed verbatim and translated (where necessary) into English. Translations focused on capturing meaning and therefore represent close approximations of interviewees’ dialogue. Table 1 provides further details of data collection.

**Table 1:** Data Collection

	Interviews	Focus groups	Questionnaires
Germany	14	4	5
Hungary	10	4	5
Ireland	13	3	5
Portugal	10	1	5
Observations at implementation team meetings		6 meetings’ fieldnotes	
Synergistic effects workshop		1 (workpackage leads and intervention site researchers)	
Total data collection		47 Interviews; 12 focus groups; 6 meetings observations/fieldnotes; 1 workshop	

Analysis of interview and focus group transcripts were conducted by one researcher (FH) and verified through team discussions where transcripts were independently analysed by MM and ROC. Qualitative analysis drew on the framework technique [15], which involves a process of charting and summarising data and distilling it into major themes, which are then used to develop an analytical matrix. The matrix noted themes and summary points using colour coding linked to the phase of data collection in order to highlight the longitudinal element of the analysis. Each intervention site was treated as a ‘case’ and within case analysis was conducted by use of framework matrices. A cross case comparison was then made by charting common themes and identifying key mechanisms of action. Although analysis of large qualitative datasets would commonly use computer assisted qualitative analysis software (CAQDAS), we felt that charts and matrices developed within Microsoft Word better facilitated a longitudinal case study analysis. Indeed we initiated analysis using QSR NVivo (v9) to organise and code material, but found that the propensity of the software to chop up transcripts into individual quotes made it very difficult to maintain an overall view of the cases and change over time. The framework analysis technique is thus particularly valuable when applied to disparate case study data as it facilitates cross case comparison.

Analysis of the first two waves of data (by FH and MM) for the process evaluation revealed that there were synergies between different components or levels of interventions. We developed a matrix (see Table 2) in which each intervention level was broken down into constituent parts and then, informed by existing evidence of synergistic interactions, we generated hypotheses about further potential synergies. The matrix was then verified by presentation to the OSPI intervention sites at a workshop with OSPI intervention country PI’s and researchers. These hypothetical synergies were explored in the subsequent waves of data collection where our sample and resources allowed. However, analysis of the full process evaluation dataset identified another important phenomenon that illustrated another way in which ‘added value’ was accrued to planned activity. This was the unanticipated impact of OSPI interventions or the programme as a whole acting as a catalyst to encourage suicide prevention activities that were external to OSPI or otherwise adding value to planned activities. Thus we illustrate below the QuaSIC approach that revealed both synergistic and catalytic interactions related to the OSPI suicide prevention programme.

**Table 2:** Multi-level intervention framework

Level 1: TARGETING PRIMARY CARE	Hypothesised synergies
Training & Train-The-Trainer (TTT) sessions for primary care health professionals	
Telephone helpline for GPs & online help for GPs and other health professionals	
Information brochures & educational videos/DVDs for GPs	
Level 2: PUBLIC HEALTH CAMPAIGN (including launch/events advertising OSPI)	
Involvement of well-known patron	Raises profile of OSPI & attracts media interest May increase help-seeking by ‘normalising’ mental health issues (impact on Level 4)
Opening ceremony/public launch	Raises public awareness of OSPI/public health campaign Publicise training & increase uptake (synergy with levels 1&3 training) Increase mental health awareness/literacy Increase media reporting Establish relationships across sectors related to suicide prevention
Launch for GPs	Raise awareness of suicide prevention Increase uptake of GP training
Press conference, local newspaper articles about OSPI	Increase uptake of media guidelines for responsible reporting of suicide Increase media reporting of OSPI activities
Public information events (e.g. depression day, jogging against depression)	Increase mental health literacy Increase help seeking
Cinema and/or radio spot	Raise awareness of OSPI activities and increase uptake of training (levels 1&3), increase help seeking (level 4)
Presentations about depression in colleges/schools	Improve mental health literacy Increase help seeking (level 4) Increase uptake of community facilitator training for teachers (level 3)
Flyers, leaflets, brochures, posters, billboards, information CDs/DVDs & promotional gifts	Improve mental health literacy Increase training uptake, help-seeking
LEVEL 3: COMMUNITY FACILITATORS’ TRAINING	
Training & TTT sessions	
Information seminars	
Media guidelines & workshops for journalists	Increase reporting of OSPI activity (level 2)
LEVEL 4: SUPPORT FOR PATIENTS AND FAMILIES	
Support for self-help groups for depression, bereaved relatives, family members of people who self-harm	
Information videos/DVDs for high risk groups & patients	
Information material/signposting (e.g. helpline numbers)	
Emergency cards for high risk suicide attempters	
Public lectures for patients and relatives	
Online patient forum	
LEVEL 5: INTERVENTIONS RELATED TO METHODS OF SUICIDE OR RESTRICTION OF ACCESS	
Provision of information at suicide hot spots (e.g. emergency telephone number)	
During CME courses: warning re TCA and other drugs with toxicity on overdose	
Disposal of Unused Medication Properly (DUMP) project	

## Results

We focus on some of the most illuminating examples from our analysis, to illustrate both synergistic and catalytic interactions. While we were able to anticipate synergies between the public health campaign and other levels of the intervention programme, many of the synergies were in fact not anticipated or hypothesised in advance (see Table 2).

### Synergistic interactions

Within the public information campaign (level 2) in both Ireland and Germany there was evidence that by inviting members of the press to attend the public launch event to advertise the initiation of OSPI activities, media interest was developed at an early stage, which in turn enhanced subsequent press coverage. As Table 3 illustrates, while Ireland had only two public information events (the least number of all four countries), there were 20 separate media reports of OSPI activity, which represents proportionately greater coverage for lower levels of activity. This is in contrast to Portugal, where there were nine public events but only 4 media reports related to OSPI activity.

**Table 3:** Cross-country comparison of intervention activity

Intervention	Germany	Hungary	Ireland	Portugal
Media coverage of OSPI activity (reports in newspapers, tv, online, radio)	64 items/reports	13 items/reports	20 items/reports	4 items/reports
Public information events (including public launch ceremony)	46	10	2	9

Fieldnotes recorded that in Ireland, a good relationship established with journalists attending the public launch of OSPI-Ireland facilitated receptivity to OSPI press releases and coverage of local suicide prevention activities. Initial media interest also prompted journalists to register for training in appropriate reporting of suicidal acts (Level 3, community facilitator training) and editors became more receptive to cascading media guidelines for responsible reporting. Thus the level 2 intervention (A) interacted with the level 3 intervention (B) to enhance the latter. Therefore, the total benefits of A + B included synergistic interactions (Sx) so that A + B + Sx > A + B.

Synergies were also detected between more than two levels of intervention. For instance, in Germany we found that the support for self-help groups for people living with or affected by depression (classified as Level 4 interventions) interacted with both the public health campaign (Level 2) and GP training (Level 1). As members of these self-help groups learned more about the aims and objectives of OSPI they (and others with an interest in depression) evolved into an active group of OSPI volunteers. These volunteers increased capacity for the public health campaign by distributing flyers, helping with public events and other activities that assisted the OSPI team. This is clearly illustrated by comparing the public information events hosted by each country (see Table 3): Germany achieved by far the highest number of events (*n* = 46), with Hungary hosting ten events, Portugal hosting nine events and Ireland hosting two. Furthermore, as the volunteer group gained momentum in supporting the public health campaign, they began to identify opportunities to disseminate campaign materials and produced ideas for events that would further the aims of the campaign. As one of the volunteers elaborated:

*Well, from the view of the* […] *volunteers, I think that many activities came from them where we had the feeling that we should reach the public at certain events or places. The materials that were provided by* [OSPI] *were very helpful. And also our contribution: watching what goes on in the city, where people meet and identifying where we can use these events and materials. That’s important, I think. Also talks,* […] *all these events were publicised by posters and flyers or the newsletter* [that we distributed] (Germany Int 5-3).

In addition to the synergies between support for self-help groups and the public health campaign, there were additional benefits from volunteer activity. Their increased visibility at public events about depression where some members spoke as patient advocates or related their experiences of depression actually led to raising awareness and interest in the self-help groups, thereby increasing help seeking behaviour. As one of the OSPI team commented:

*They support us, they do administrative work, they organise things, they feel that things are changing positively. They also say there are more people now expressing interest to join self-help groups* (Germany Int 8-3).

Feedback from the German self help group/volunteers also illustrates evidence of a synergistic interaction between Level 4 (support for patients and families) and Level 1 (training for GP’s). One member of a volunteer group recruited her GP to primary care training through her enthusiastic dissemination of OSPI activities during a consultation.

Respondent: *I know that my GP, to whom I always bring the self-help magazine and also the* [OSPI] *flyers, was very happy and open about the offer of training for GPs. Actually, she got to know about these activities from me.*

Researcher: *Do you know if she participated in a training session?*

Respondent: *Yes, yes, at one of the very first* (Germany Int 5-3).

The benefits or impacts of the three different levels of intervention were enhanced by the synergistic interactions between them. This is represented by the formula A + B + C + Sx > A + B + C.

### Catalytic impacts from interventions

In addition to synergies within or between levels of interventions, there are also catalytic interactions. These occur when single levels of intervention or indeed the whole programme, acts as a catalyst to stimulate related activity implemented by those individuals or agencies that are external to the intervention teams. In particular, catalytic interactions generated by the implementation activity produced two different kinds of impacts. Firstly, they generated something new that fed back into and enhanced planned activities, or secondly, stimulated additional, external activity with the shared goal of suicide prevention.

For example, the OSPI team in Portugal found that initiating suicide prevention training and rolling out the public awareness campaign in their intervention region stimulated complimentary activities developed by professionals with a shared interest in suicide prevention. Subsequent to OSPI suicide prevention and awareness training with health and community professionals, a local psychiatrist took the initiative to provide similar training within his hospital. In a qualitative interview, he revealed that OSPI had had the effect of putting suicide prevention ‘*on the radar’*. Thus the additional training initiated by professionals external to the OSPI team added value to the shared goal of suicide prevention. Similarly, in Hungary the public awareness campaign (in particular the social marketing spots in local cinemas) stimulated local interest in suicide prevention, highlighted the need for more mental health infrastructure and acted as a catalyst for local action and increased investment/resource. This led to the planned development of a new mental health drop in centre in the intervention region. Unfortunately, towards the end of the implementation period the impact of the recession became such that resources initially allocated to this project were no longer available, so this new resource was not implemented. However, this does illustrate the potential for a programme to act as a catalyst to stimulate external interest in contributing to shared goals (in this case, suicide prevention).

In Hungary, a focus group participant revealed how involvement in OSPI activities had the unanticipated consequence of improving communication between professional groups: ‘*the OSPI programme gave a great impetus for psychiatrists and GPs to get together. This contact has been established, and psychiatrists and GPs now talk to each other’* (Hungary FG1-3). A potential benefit of this improved communication was improvements in referral pathways and access to specialist support, potentially furthering OSPI’s suicide prevention goals. Furthermore, interdisciplinary training sessions in both Ireland and Hungary widened both personal and organisational networks that enhanced cross-sector collaborations and referral pathways.

*And again I suppose maybe not just even dealing with serious self-harm or that stuff, but when they meet other people from other agencies, they make contacts in relation to other matters as well, you know. If there’s a query that maybe the HSE, the Health Service Executive (HSE), can help us with, they have a contact they can go to straight away, and also the HSE have a contact with the* [police force]. *So eventually it will build up a wider network* (Ireland Int 3-1).

Thus, catalytic interactions can be conceptualised as P (OSPI Programme) → (generates) x, y and z; where the latter are activities external to OSPI but nevertheless share similar programme goals.

## Discussion

Our data reveal hidden additional benefits of a complex intervention through exploring synergistic interactions between intervention levels, as well as the catalytic interactions that generated external activity sharing similar goals in suicide prevention. Making the distinction between synergies and catalysts illustrates the further potential for maximising suicide prevention activities. We thus arrive at a refined definition:

1. *Synergistic interactions in complex interventions (either single or multi-level) achieve an impact that is greater than the sum of effects of interventions provided in isolation.*
2. *Catalytic interactions in complex interventions (either single or multi-level) are those that stimulate additional activity that add value to, but are nevertheless external to, planned activities.*

Understanding the potential synergistic and catalytic interactions in complex interventions is crucial to maximising the potential of suicide prevention and indeed other complex programmes. A recent systematic review of suicide prevention strategies emphasised the need to explore what works within suicide prevention [5], but found no evidence of studies that explored the synergistic interactions within suicide prevention programmes. Similarly, a recent study estimating the impacts of a multi-level systems approach to suicide prevention has also concluded that potential synergistic effects between strategies could further increase their impact. However, they could only hypothesise on the potential for the combined effects from multiple interventions to have more or less of an impact on outcomes and acknowledged the need to understand the context of implementation and any regional challenges that may arise [16]. This highlights the value of our study, as it provides unique examples of such context dependent synergies across different sites. While we have demonstrated how levels interact to produce synergies that are greater than the sum of the two (or more) parts, we have also illustrated the added value accrued to a suicide prevention programme through additional, external activities that were stimulated by association. Interestingly, the most complex synergies between support for patients and families, GP training and the public health campaign were not hypothesised at the outset and yet if we had not set out to explore synergistic interactions within our data, these interactions may have remained hidden. Understanding more about synergistic and catalytic interactions may facilitate planning to maximise the contexts conducive to these synergies. For instance, our data suggest the following recommendations for optimising the OSPI multi-level suicide prevention approach:

- engage service user groups and local volunteers as collaborators where possible and co-ordinate volunteer activities to maximise the added capacity for implementation activity
- time the public launch to be close to the delivery of implementation activity to maximise the potential synergies between media reporting, media interest, take-up of public awareness campaign messages, recruitment to training and so on.

We were able to identify additional interventions initiated by external agencies in response to the catalytic action of OSPI. Since the external activity (such as additional suicide prevention training in a hospital in Portugal) will also potentially contribute to improved outcomes such as reductions in suicidal behaviour, it is crucial that evaluations take note of this. By exploring the additional activity that is generated by catalytic interaction, funders can also gain a greater understanding of aspects of a complex intervention that usually remain hidden, but nevertheless may represent substantial added value if factored into a model of benefits and costs.

### Limitations of the study

What the QuaSIC approach cannot do is provide a measure of effect, based as it is on qualitative methods. However, we would question whether in fact it is possible to establish a quantitative measure of effect when the interactions between interventions are of such a complex nature. This is particularly the case where interventions are implemented in locations where previous health promoting programmes may already have generated conditions for synergy, by for instance already fostering good links between researchers, policy makers, practitioner and the media. This is also the case for catalytic interactions, where it would be almost impossible to quantify the effect size due to ‘spin off’ activity.

Another important limitation is that we did not consider the possibility that rather than just creating synergies there may in fact be adverse consequences that arise from complex interventions that reduce their overall effectiveness [3]. Within a multi-level intervention, actions at one level may be crowded out by factors at other levels that mediate the relationship between the intervention and intended outcome as has been the case with some programmes to promote healthy eating in schools [17]. In respect of external impacts, it may also be possible that a new complex programme could crowd out rather than complement existing actions and programmes. This may be particularly the case if existing and new programmes have to compete for the same set of resources. While information to date is limited, these negative impacts have sometimes been documented following the implementation of HIV protection programmes in some countries [18, 19]. However, determining whether such adverse effects may take place can only be made some time after a new programme is developed and, in the case of OSPI, after initial funding provided as part of a research programme has been exhausted. Longer term follow up is required to determine what positive and/or negative synergies may arise from sustaining new programmes in a landscape where some interventions may already be in place. There are also potential impacts on other health promotion programmes, such as initiatives to promote mental health that should be considered, particularly if these are subsequently viewed as lower priorities for support.

## Conclusions

We have identified the importance of exploring synergistic and catalytic interactions in complex, multi-level interventions using the QuaSIC approach. It is important to hypothesise (or somehow identify) anticipated interactions and then engage in systematic data collection processes which can help evidence whether these occur and under what circumstances. Synergies can occur both within and across levels as multiple activities are often required to implement different levels of activity. Either the whole programme of activity or single levels of intervention can act as a catalyst to generate unanticipated, additional effects that may also affect outputs/outcomes. Future research should also explore potential negative synergies and how to mediate or minimise these.

Our process evaluation paid close attention to the contexts of implementation, thus we identified the complex interplay between the intervention elements, levels and external activity. We surmise that it is largely due to sharing the goal of suicide prevention with local communities that the opportunity for synergies and additional impacts were maximised. Furthermore, rather than imposing radically new interventions, OSPI activities were complementary to existing local programmes, limiting the risk of any negative synergies and thereby paving the way to adding value to what we hope will be a reduction of both non-fatal suicidal acts and deaths by suicide.

## Informed consent

Written informed consent was obtained from all participants for publication of this research article. A copy of the written consent is available for review by the Editor of this journal.

### Data availability

The data for this paper is not publicly available due to the fact that interviewees and focus group participants were key stakeholders in suicide prevention and could potentially be identifiable by role alone. Quotes were carefully selected to protect confidentiality and to ensure that they were non-attributable. Interviewees consented to data only being shared with the research team.
